# The cardiac enigma: current conundrums in heart failure research

**DOI:** 10.12688/f1000research.7278.1

**Published:** 2016-01-18

**Authors:** Michael S. Kapiloff, Craig A. Emter

**Affiliations:** 1 Cardiac Signal Transduction and Cellular Biology Laboratory, Interdisciplinary Stem Cell Institute, Departments of Pediatrics and Medicine, Leonard M. Miller School of Medicine, University of Miami, Miami, FL, USA; 2 Department of Biomedical Sciences, University of Missouri-Columbia, Columbia, MO, USA

**Keywords:** Heart failure, clinical trials, angiotensin-converting enzyme

## Abstract

The prevalence of heart failure is expected to increase almost 50% in the next 15 years because of aging of the general population, an increased frequency of comorbidities, and an improved survival following cardiac events. Conventional treatments for heart failure have remained largely static over the past 20 years, illustrating the pressing need for the discovery of novel therapeutic agents for this patient population. Given the heterogeneous nature of heart failure, it is important to specifically define the cellular mechanisms in the heart that drive the patient’s symptoms, particularly when considering new treatment strategies. This report highlights the latest research efforts, as well as the possible pitfalls, in cardiac disease translational research and discusses future questions and considerations needed to advance the development of new heart failure therapies. In particular, we discuss cardiac remodeling and the translation of animal work to humans and how advancements in our understanding of these concepts relative to disease are central to new discoveries that can improve cardiovascular health.

## Introduction

Heart failure, the common end stage of heart disease, is defined clinically by fatigue, shortness of breath, and fluid retention, including pulmonary edema ^1^. Heart failure is a syndrome of major public health significance, impacting 5.7 million worldwide with an incidence of 870,000 adults in the United States alone. The prevalence of heart failure is expected to increase by 46% by 2030, and this is due in large part to aging of the general population but also to the improved survival following events such as myocardial infarction and the increased prevalence of comorbidities such as obesity and diabetes ^2^. The cost to society is consequential. The 5-year mortality for heart failure remains approximately 50%, despite current therapies. Moreover, the financial costs associated with heart failure are expected to balloon to over $70 billion per year by 2030 ^1^. As a result, the discovery of new drug targets for heart failure prevention or treatment (or both) remains an area of pressing concern.

## The current approach to chronic heart failure therapy

Current therapies for heart failure are both medicinal and device-driven ^3^. The mainstays of heart failure pharmacotherapy include β-blockers (β-adrenergic receptor antagonists such as carvedilol, metoprolol, and bisoprolol), angiotensin-converting enzyme inhibitors ([ACEI] e.g., enalapril and lisinopril), angiotensin II receptor blockers (e.g., losartan and valsartan), aldosterone antagonists, hydralazine and isosorbide dinitrate, and diuretics ^4^. Ventricular assist devices, such as the implantable cardioverter-defibrillator, left ventricular (LV) assist device, and cardiac resynchronization therapy, are widely used, and cardiac transplant remains a therapy of last resort for some patients. There has been tremendous excitement this year as the first new drugs since 1999 have been approved by the US Food and Drug Administration for chronic heart failure. Ivabradine (Corlanor) is a sinoatrial node I _f_ current inhibitor that has been shown to have efficacy in a variety of clinical trials, including SHIFT (Systolic Heart failure treatment with the *I*_f_ inhibitor ivabradine Trial) ^5– 7^. LCZ696 (Entresto™) is a combination drug including a neprilysin endopeptidase inhibitor (sacubitril) and angiotensin receptor blocker (valsartan). In the PARADIGM-HF (Prospective Comparison of ARNI With ACEI to Determine Impact on Global Mortality and Morbidity in Heart Failure) trial, LCZ696 was shown to reduce mortality in comparison with enalapril ^8^. Finally, ranolazine improved hemodynamic status in the proof-of-concept RALI-DHF (RAnoLazIne for the Treatment of Diastolic Heart Failure) trial ^9^. However, even with these new drugs, heart failure morbidity and mortality are expected to increase substantially in the near future.

## Heart Failure: HFrEF vs. HFpEF

In considering the future of heart failure therapies, it is important to remember that heart failure is not a homogenous entity but instead a clinical syndrome related to end-stage heart disease. Heart failure can be divided into two groups: (a) reduced ejection fraction (HFrEF) and (b) preserved ejection fraction (HFpEF) (ejection fraction >45%); the two forms confer a similar prognosis and have similar prevalence ^10– 12^. As the names suggest, HFrEF includes prominent systolic cardiac dysfunction, whereas HFpEF is more closely associated with diastolic dysfunction. Although the current therapies for heart failure have been established in clinical trials involving HFrEF patients, none has been therapeutically useful for HFpEF patients ^10, 11, 13, 14^. Instead, exercise may be the only clinically effective, currently available HFpEF treatment ^15^, and several small-scale clinical trials show that exercise improves cardiorespiratory variables such as oxygen consumption (VO _2_), diastolic dysfunction, and quality of life in HFpEF patients ^16^. The Exercise Training in Diastolic Heart Failure (EX-DHF) (ISRCTN 86879094) study will examine these findings on a larger scale and hopefully provide insight regarding the use of exercise to improve mortality. The lack of drug therapies for HFpEF may be due to the limited efficacy of current drugs to treat diastolic dysfunction. Alternatively, it was recently suggested that many patients with diagnosed HFpEF, typically older women with diabetes and obesity, do not have structural heart disease but instead exhibit fairly general signs that define heart failure due to non-cardiac problems ^17^. For example, although pulmonary edema is usually a reliable indicator of LV dysfunction (and high diastolic pressures), it can also be caused by primary pulmonary disease (e.g., adult respiratory distress syndrome) or low plasma colloid oncotic pressure due to other etiologies ^18^. HFpEF should also be distinguished from high-output heart failure, a form of heart failure that typically is secondary to a non-cardiac disease ^19^. In high-output heart failure, the heart responds normally and reversibly to extra-cardiac stress, often undergoing physiologic hypertrophy that is induced by low afterload and volume overload on the heart. Thus, when novel approaches to the treatment of heart failure are considered, it is important to precisely define the cohort of patients being considered and whether the heart is in fact the relevant target for therapeutic intervention.

## Pathological cardiac remodeling

A long-term goal of heart failure therapies is to reverse or prevent cardiac remodeling, the general term referring to the structural changes in the heart induced by chronic stress. While the heart can respond to acute demands for increased output by changes in chronotropy (heart rate), inotropy (contractility), and lusitropy (relaxation), the adult heart is generally limited to hypertrophy as a compensatory mechanism ^20^. Cardiac hypertrophy at the whole-organ level reflects non-mitotic growth of the cardiac myocytes. The generally cylindrical adult myocyte can grow in either width (diameter) or length, resulting in thickened ventricular walls or chamber dilation, respectively. In theory, concentric myocyte growth increases the width of cardiomyocytes, inducing parallel assembly of sarcomeres and thereby reducing ventricular wall stress (Laplace’s law). In contrast, eccentric myocyte growth increases cardiomyocyte length, inducing serial addition of sarcomeres to accommodate greater ventricular volumes without stretching individual sarcomeres beyond the optimum length for contraction (Frank-Starling law) ^21^. In pressure overload diseases, such as aortic stenosis or hypertension, there is increased systolic wall stress, and concentric hypertrophy initially predominates. In volume overload diseases, such as following a myocardial infarction or dilated cardiomyopathy, eccentric hypertrophy predominates, presumably in response to increased diastolic wall stress. Although sarcomeric assembly is considered initially compensatory, myocyte hypertrophy is eventually concomitant with altered myocyte gene expression, metabolism, excitation-contraction coupling, increased cell death, and myocardial fibrosis. Together, these factors contribute to systolic and diastolic cardiac dysfunction and promote pathological cardiac remodeling and subsequent heart failure.

## Outstanding questions in heart failure research

The above description of heart failure is the basis for the underlying paradigm driving most research in the field, primarily the identification of potential drug targets that protect cardiac contractility and inhibit myocyte death and interstitial fibrosis ^22, 23^. We propose the following issues as central to advancing the treatment of heart failure, including questions to stimulate discussion about the underlying assumptions concerning disease development:

1. A fundamental question concerns whether any of the features in cardiac remodeling are necessarily compensatory (i.e., can be safely targeted in the face of cardiac stress). For example, as recently discussed in a point-counterpoint editorial series in *Circulation* ^24, 25^, concentric LV hypertrophy is often considered compensatory in diseases of increased afterload. The current first-line therapies for heart failure target the adrenergic and renin-angiotensin systems, having effects both on cardiac myocytes and on the vasculature ^25^. It has been argued that lowering afterload and LV wall stress is essential to the efficacy of these drugs in patients (whether by lowering blood pressure for hypertension or by contemporaneous aortic valve replacement for aortic stenosis) and that attenuating hypertrophy without lowering afterload would not be tolerated in humans ^26^. However, diverse studies in rodents using pharmacological agents such as cyclosporine, and more elegantly with genetically modified mice, have shown that inhibition of hypertrophy not only is tolerated in the face of persistent pressure overload but also can prevent or treat heart failure ^22^.

2. It remains unclear which features in cardiac remodeling are co-regulated by signal pathways that may be targeted and which features may be specifically targeted independently of other aspects of remodeling. Although most studies show that hypertrophy and fibrosis are tightly associated in disease, recent findings by the Backs laboratory showed that mice lacking the γ and δ isoforms of Ca ^2+^/calmodulin-dependent protein kinase II had improved cardiac function and decreased fibrosis but persistent hypertrophy following transverse aortic constriction ^27^. Whereas some aspects of the changes in myocyte gene expression, metabolism, and excitation-contraction coupling may be detrimental (e.g., the decreased sarcoplasmic reticulum Ca ^2+^ ATPase [SERCA2a] activity in heart failure), others may be beneficial (e.g., increased natriuretic peptide expression or concentric hypertrophy that decreases wall stress). These exciting findings suggest that the beneficial aspects of remodeling may be retained while therapeutically combatting the deleterious aspects that lead to heart failure.

3. It remains unclear what causes diastolic dysfunction, especially in HFpEF. Diastolic dysfunction is the result of reduced active relaxation or ventricular compliance. Active relaxation occurs in large part due to ATP-dependent Ca ^2+^ reuptake during diastole primarily through SERCA2a, and reduced SERCA2a activity is associated with heart failure. Accordingly, in animal models, SERCA2a replacement has been effective in improving overall cardiac function ^28^. Decreased compliance and associated atrial hypertrophy have long been associated with interstitial fibrosis ^29, 30^. However, the relative extent of fibrosis and altered Ca ^2+^ reuptake can vary in different models for diastolic dysfunction ^30, 31^, raising the question of what should be targeted in diastolic dysfunction under different clinical scenarios. In numerous animal models, reversal or reduction of LV hypertrophy has been shown to improve diastolic function independently of hemodynamic alteration, implying that hypertrophy itself plays a role in diastolic dysfunction. However, clinical studies have shown that this relationship is less apparent in humans ^32^. Finally, coronary vascular dysfunction may have a profound impact on myocardial oxidative capacity and diastolic dysfunction in heart failure ^33^. Recent work has shown that swine with diastolic dysfunction have myocardial oxygen supply/demand imbalance ^34^. This suggests that impaired coronary vasculature function may contribute to the inability of HFpEF patients to respond to situations of increasing stress by limiting ATP production and subsequent active relaxation.

4. Humans are not large mice. Little is known or being investigated about cardiac signal transduction in large mammals, despite the significant differences between large and small mammalian hearts. Instead, most of what is known about the regulation of myocyte hypertrophy and cardiac remodeling has been defined in mice and rats ^25^. Differences between large and small mammals include life span, heart rate, excitation-contraction coupling and Ca ^2+^ handling, α:β-myosin heavy chain ratio, tolerance for myocardial injury, and rate of progression of cardiac remodeling ^25^. The successful development of therapeutics for human patients is dependent upon the identification of mechanisms that are in fact relevant to the large mammalian heart. For example, many question whether large animals subject to pressure overload can tolerate diminished hypertrophy similar to mice ^24^. A review by Dixon and Spinale discusses the importance of large animal models in translating basic science findings to the clinic and addresses the lack of studies using large animals to address pressure overload LV hypertrophy and its role in the development of heart failure ^35^. An advantage of large animal models is that key determinants of myocardial work and energy consumption, including LV wall tension, heart rate, and vascular wall-to-lumen ratios ^36^, are similar to those in humans. Thus, large animal models could provide an essential link to implement discoveries made in murines into models exhibiting functional and anatomical similarities more analogous to humans as a means to assess therapeutic potential for treating heart failure clinically. Significant financial challenges exist in generating, sustaining, and implementing large animal models of heart failure into research programs. Specific programs aimed at soliciting and supporting large animal cardiovascular research from both federal and private sources would help stimulate more large animal studies in the future and aid in bridging the gap between small animals, large animals, and humans.

## Hope for the future

It is an exciting time to be involved in heart failure research. There are ample animal models, both small and large, addressing diverse types of heart diseases, as well as protocols to study cardiac cell types *in vitro*. Our knowledge of cardiac cell regulation continues to rapidly increase, and new tools, including novel methods for visualizing signaling in real time, are being developed ^37^. In addition, adeno-associated viruses are emerging as a viable therapeutic approach to deliver both small interfering RNA (siRNA) and proteins *in vivo* for both scientific and clinical purposes ^38^. These advances portend the discovery of additional cardiac therapeutics in the coming years.

## References

[1] Mozaffarian D Benjamin EJ Go AS: Heart disease and stroke statistics--2015 update: a report from the American Heart Association. *Circulation.* 2015;131(4):e29–322. 10.1161/CIR.0000000000000152 25520374

[2] Heidenreich PA Albert NM Allen LA: Forecasting the impact of heart failure in the United States: a policy statement from the American Heart Association. *Circ Heart Fail.* 2013;6(3):606–19. 10.1161/HHF.0b013e318291329a 23616602 PMC3908895

[3] Yancy CW Jessup M Bozkurt B: 2013 ACCF/AHA guideline for the management of heart failure: executive summary: a report of the American College of Cardiology Foundation/American Heart Association Task Force on practice guidelines. *Circulation.* 2013;128(16):1810–52. 10.1161/CIR.0b013e31829e8807 23741057

[4] Xie M Burchfield JS Hill JA: Pathological ventricular remodeling: therapies: part 2 of 2. *Circulation.* 2013;128(9):1021–30. 10.1161/CIRCULATIONAHA.113.001879 23979628 PMC3829603

[5] Komajda M Tavazzi L Francq BG: Efficacy and safety of ivabradine in patients with chronic systolic heart failure and diabetes: an analysis from the SHIFT trial. *Eur J Heart Fail.* 2015;17(12):1294–301. 10.1002/ejhf.347 26377342

[6] Rogers JK Kielhorn A Borer JS: Effect of ivabradine on numbers needed to treat for the prevention of recurrent hospitalizations in heart failure patients. *Curr Med Res Opin.* 2015;31(10):1903–9. 10.1185/03007995.2015.1080155 26361063

[7] Borer JS Böhm M Ford I: Efficacy and safety of *ivabradine* in patients with severe chronic systolic heart failure (from the SHIFT study). *Am J Cardiol.* 2014;113(3):497–503. 10.1016/j.amjcard.2013.10.033 24332674

[8] McMurray JJ Packer M Desai AS: Angiotensin-neprilysin inhibition versus enalapril in heart failure. *N Engl J Med.* 2014;371(11):993–1004. 10.1056/NEJMoa1409077 25176015

[9] Maier LS Layug B Karwatowska-Prokopczuk E: RAnoLazIne for the treatment of diastolic heart failure in patients with preserved ejection fraction: the RALI-DHF proof-of-concept study. *JACC Heart Fail.* 2013;1(2):115–22. 10.1016/j.jchf.2012.12.002 24621836

[10] Borlaug BA Paulus WJ: Heart failure with preserved ejection fraction: pathophysiology, diagnosis, and treatment. *Eur Heart J.* 2011;32(6):670–9. 10.1093/eurheartj/ehq426 21138935 PMC3056204

[11] Maeder MT Kaye DM: Heart failure with normal left ventricular ejection fraction. *J Am Coll Cardiol.* 2009;53(11):905–18. 10.1016/j.jacc.2008.12.007 19281919

[12] Hunt SA Abraham WT Chin MH: ACC/AHA 2005 Guideline Update for the Diagnosis and Management of Chronic Heart Failure in the Adult: a report of the American College of Cardiology/American Heart Association Task Force on Practice Guidelines (Writing Committee to Update the 2001 Guidelines for the Evaluation and Management of Heart Failure): developed in collaboration with the American College of Chest Physicians and the International Society for Heart and Lung Transplantation: endorsed by the Heart Rhythm Society. *Circulation.* 2005;112(12):e154–235. 10.1161/CIRCULATIONAHA.105.167586 16160202

[13] Paulus WJ van Ballegoij JJ: Treatment of heart failure with normal ejection fraction: an inconvenient truth! *J Am Coll Cardiol.* 2010;55(6):526–37. 10.1016/j.jacc.2009.06.067 20152557

[14] Desai AS: Heart failure with preserved ejection fraction: time for a new approach? *J Am Coll Cardiol.* 2013;62(4):272–4. 10.1016/j.jacc.2013.03.075 23684678

[15] McDonald KS Emter CA: Exploring new concepts in the management of heart failure with preserved ejection fraction: is exercise the key for improving treatment? *J Appl Physiol (1985).* 2015;119(6):724–5. 10.1152/japplphysiol.00570.2015 26229001

[16] Dieberg G Ismail H Giallauria F: Clinical outcomes and cardiovascular responses to exercise training in heart failure patients with preserved ejection fraction: a systematic review and meta-analysis. *J Appl Physiol (1985).* 2015;119(6):726–33. 10.1152/japplphysiol.00904.2014 25749444

[17] Sharma K Kass DA: Heart failure with preserved ejection fraction: mechanisms, clinical features, and therapies. *Circ Res.* 2014;115(1):79–96. 10.1161/CIRCRESAHA.115.302922 24951759 PMC4146618

[18] Stein L Beraud JJ Morissette M: Pulmonary edema during volume infusion. *Circulation.* 1975;52(3):483–9. 10.1161/01.CIR.52.3.483 1157248

[19] Anand IS Florea VG: High Output Cardiac Failure. *Curr Treat Options Cardiovasc Med.* 2001;3(2):151–9. 11242561 10.1007/s11936-001-0070-1

[20] Hill JA Olson EN: Cardiac plasticity. *N Engl J Med.* 2008;358(13):1370–80. 10.1056/NEJMra072139 18367740

[21] Grossman W Jones D McLaurin LP: Wall stress and patterns of hypertrophy in the human left ventricle. *J Clin Invest.* 1975;56(1):56–64. 10.1172/JCI108079 124746 PMC436555

[22] van Berlo JH Maillet M Molkentin JD: Signaling effectors underlying pathologic growth and remodeling of the heart. *J Clin Invest.* 2013;123(1):37–45. 10.1172/JCI62839 23281408 PMC3533272

[23] Burchfield JS Xie M Hill JA: Pathological ventricular remodeling: mechanisms: part 1 of 2. *Circulation.* 2013;128(4):388–400. 10.1161/CIRCULATIONAHA.113.001878 23877061 PMC3801217

[24] Crozatier B Ventura-Clapier R: Inhibition of hypertrophy, per se, may not be a good therapeutic strategy in ventricular pressure overload: other approaches could be more beneficial. *Circulation.* 2015;131(16):1448–57. 10.1161/CIRCULATIONAHA.114.013895 25901070

[25] Schiattarella GG Hill JA: Inhibition of hypertrophy is a good therapeutic strategy in ventricular pressure overload. *Circulation.* 2015;131(16):1435–47. 10.1161/CIRCULATIONAHA.115.013894 25901069 PMC4408778

[26] Drazner MH: The progression of hypertensive heart disease. *Circulation.* 2011;123(3):327–34. 10.1161/CIRCULATIONAHA.108.845792 21263005

[27] Kreusser MM Lehmann LH Keranov S: Cardiac CaM Kinase II genes δ and γ contribute to adverse remodeling but redundantly inhibit calcineurin-induced myocardial hypertrophy. *Circulation.* 2014;130(15):1262–73. 10.1161/CIRCULATIONAHA.114.006185 25124496 PMC4316667

[28] Kho C Lee A Hajjar RJ: Altered sarcoplasmic reticulum calcium cycling--targets for heart failure therapy. *Nat Rev Cardiol.* 2012;9(12):717–33. 10.1038/nrcardio.2012.145 23090087 PMC3651893

[29] Villari B Campbell SE Hess OM: Influence of collagen network on left ventricular systolic and diastolic function in aortic valve disease. *J Am Coll Cardiol.* 1993;22(5):1477–84. 10.1016/0735-1097(93)90560-N 8227808

[30] Ishikawa K Aguero J Oh JG: Increased stiffness is the major early abnormality in a pig model of severe aortic stenosis and predisposes to congestive heart failure in the absence of systolic dysfunction. *J Am Heart Assoc.* 2015;4(5): pii: e001925. 10.1161/JAHA.115.001925 25994443 PMC4599422

[31] Hiemstra JA Gutiérrez-Aguilar M Marshall KD: A new twist on an old idea part 2: cyclosporine preserves normal mitochondrial but not cardiomyocyte function in mini-swine with compensated heart failure. *Physiol Rep.* 2014;2(6): pii: e12050. 10.14814/phy2.12050 24963034 PMC4208639

[32] Heinzel FR Hohendanner F Jin G: Myocardial hypertrophy and its role in heart failure with preserved ejection fraction. *J Appl Physiol (1985).* 2015;119(10):1233–42. 10.1152/japplphysiol.00374.2015 26183480 PMC4652334

[33] Heinonen I Sorop O de Beer VJ: What can we learn about treating heart failure from the heart's response to acute exercise? Focus on the coronary microcirculation. *J Appl Physiol (1985).* 2015;119(8):934–43. 10.1152/japplphysiol.00053.2015 26048972

[34] Marshall KD Muller BN Krenz M: Heart failure with preserved ejection fraction: chronic low-intensity interval exercise training preserves myocardial O _2_ balance and diastolic function. *J Appl Physiol (1985).* 2013;114(1):131–47. 10.1152/japplphysiol.01059.2012 23104696 PMC3544520

[35] Dixon JA Spinale FG: Large animal models of heart failure: a critical link in the translation of basic science to clinical practice. *Circ Heart Fail.* 2009;2(3):262–71. 10.1161/CIRCHEARTFAILURE.108.814459 19808348 PMC2762217

[36] Douglas WR: Of pigs and men and research: a review of applications and analogies of the pig, sus scrofa, in human medical research. *Space Life Sci.* 1972;3(3):226–34. 10.1007/BF00928167 4556756

[37] Sprenger JU Nikolaev VO: Biophysical techniques for detection of cAMP and cGMP in living cells. *Int J Mol Sci.* 2013;14(4):8025–46. 10.3390/ijms14048025 23584022 PMC3645729

[38] Zacchigna S Zentilin L Giacca M: Adeno-associated virus vectors as therapeutic and investigational tools in the cardiovascular system. *Circ Res.* 2014;114(11):1827–46. 10.1161/CIRCRESAHA.114.302331 24855205

